# The global regulatory landscape for combined vaccines: A comparative case study of registration strategies for diphtheria-tetanus-pertussis-containing vaccines.

**DOI:** 10.1016/j.vaccine.2025.127017

**Published:** 2025-04-30

**Authors:** Xiangchuan He, Yiwen Pu, Zeyu Li, Shitong Huan, Yue Yang

**Affiliations:** a School of Pharmaceutical Sciences, Tsinghua University, Beijing 100084, China; b Key Laboratory of Innovative Drug Research and Evaluation, National Medical Products Administration, Beijing 100084, China; c The Scientific and Technological Achievement Transformation Center, Beijing Youan Hospital, Capital Medical University, Beijing, 100069, China; d China office, The Bill & Melinda Gates Foundation, Beijing, 100084, China

## Abstract

Diphtheria-tetanus-pertussis-containing vaccines (DTPCVs) represent the forefront of combination vaccine development globally. However, the licensing of high-valent DTPCVs varies worldwide owing to diverse regulatory requirements. Some national regulatory authorities (NRAs) have adopted flexible strategies to fast-track the approval and uptake of critical DTPCVs to address public health needs. This study examined the global regulatory status of combination vaccines, using descriptive and comparative analyses to highlight pivotal registration issues for DTPCVs. Specifically, it sheds light on the regulatory approaches of the Food and Drug Administration (FDA), European Medicines Agency (EMA), National Medical Products Administration (NMPA), and other Asian regulators through a case study of hepatitis B-containing DTPCVs. Drawing on these findings, this study advocates the initiation of regulatory convergence efforts, establishment of practical regulatory strategies, and swift approval and wider adoption of high-valent DTPCVs.

## 1 Introduction

Over the last few centuries, substantial progress has been made in the elimination or eradication of various infectious diseases. Among the public health interventions that have proven to be effective to achieve this objective, immunization is one of the most cost-effective measures available in countries of all income levels [1]. More than 20 life-threatening diseases can now be prevented by employing vaccination, as a cornerstone of health policy [2]. Combinations of vaccines can streamline processes, reduce costs, and create room for new vaccines in immunization programs, bolstering public health, economic efficiency, and societal well-being by enhancing vaccine coverage and compliance [3,4]. Thus, combination vaccines represent an important focal point of innovation in vaccine research and development.

The diphtheria-, tetanus- and pertussis containing vaccine (DTPCV), measles containing vaccine (MCV), and other combination vaccines represent the three main strategies for developing combination vaccines, Among theses, DTPCV has the highest number of components, offering up to 12 product categories and more than 10 components [5]. Hexavalent DTPCVs (DTP-HBV-IPV-Hib) are notable for their high valence and, protection against six diseases: diphtheria (D), tetanus (T), whooping cough (P), polio, hepatitis B (HepB), and *Haemophilus influenzae* type b (Hib) infection. Children receiving this vaccine can be immunized with four doses of the hexavalent vaccine in the first two years of their life, instead of receiving more than ten shots if the vaccines are administered separately [6,7]. Additionally, DTPCVs offer the scope for adding new antigens, with ongoing research on the addition of a meningococcal component to the hexavalent formulation, representing a promising direction for future development [4,8].

Among the 194 World Health Organization (WHO) member nations, over 120 have introduced the hexavalent vaccine [9], with 63 incorporating it into their National Immunization Programs (NIPs). Nevertheless, hexavalent DTPCV is still not available in nearly 60 % of these countries,including Niger, Chad, Afghanistan, which have relatively higher birth rates [10]. Generally,gaps in combination vaccine use encompass four dimensions, legislation and regulation, immunization schedule design, vaccine awareness and pricing, and research and development (R&D) capacity [11,12].

Typically, formulation of combination vaccines, as new vaccine products, is more complex than formulating simple mixtures of several antigens [13]. The development of DTPCV combination vaccines involves some key technical aspects, including the use of antigens based on acellular or whole-cell pertussis, formulations with full liquid or reconstituted lyo-liquid, and the choice of compatible adjuvants. The WHO and several regulatory agencies have published a series of regulatory documents, including guidelines and recommendations for combination vaccines. These WHO documents form the basis for WHO prequalification and the purchase of vaccines for global immunization programs by the United Nations (UN), and can be adopted by national regulatory authorities (NRAs) and manufacturers as definitive national requirements.

For combination vaccine development, the WHO states that the clinical program will vary according to the specific combination vaccine being tested, and according to previous clinical experience with individual and similar vaccines. Moreover, the vaccine-specific requirements for clinical studies should be discussed with the appropriate NRA [14].

Despite the potential future breakthroughs to address the existing technical difficulties, conservative regulatory registration requirements for higher-valent combination vaccines in some local NRAs may still pose some challenge [11,15,16]. In China, the most prevalent domestic DTPCV is a tetravalent vaccine, with pentavalent DTPCV entering phase I clinical trials. Although an imported pentavalent DTPCV has been launched, no hexavalent DTPCV has been approved for clinical trials [11,12]. The registration issues associated with this scenario primarily involve (1) cooperative development and flexible multi-site manufacturing, (2) alignment of the immunization schedules of the vaccines to be combined, and (3) clinical evaluation of the combination vaccine under different scenarios [[16], [17], [18]].

Global experience has shown that various strategies and modalities can be employed to facilitate the registration of combination vaccines. This study compared and analyzed the differences in regulations and guidelines for registering combination vaccines among leading regulatory agencies, and explained the scientific logic and registration paths of various countries to address the challenges facing high-valent DTPCVs. Furthermore, this study used HepB-containing hexavalent vaccines to conduct a case study encompassing several representative countries. Scientific recommendations have been proposed to accelerate the registration of combination vaccines by pre-emptively identifying issues and regulatory barriers.

## 2 Materials and methods

### 2.1 Selection of the representative DTPCV products

Using insight databases (db.dxy.cn, an integrated website with real-time updates of global authoritative pharmaceutical and clinical data), we extracted the characteristcs of globally marketed DTPCVs products as of 1 January 2024, and the validated and approval data from the websites of manufacturers, including GSK, Sanofi Pasteur, MCM (a partnership between Sanofi and Merck to produce pediatric vaccines), Beijing Minhai, Panacea and the Serum Institute of India. In addition, we selected and analyzed the first licensed DTPCVs globally.

### 2.2 Search and selection strategy for regulatory documents

To better understand the regulatory considerations of combination vaccines for registration between agencies, as of 1 January 2024, we searched the WHO documents databases (https://www.who.int/publications/m) for key words of combined (or combination) vaccine recommendations, and vaccine position papers concerning to DTPCV-related components. We also searched out the regulatory documents related to combined vaccines from some main NRAs websites including FDA (https://www.fda.gov/), EMA (https://www.ema.europa.eu/en/homepage), MFDS (https://www.mfds.go.kr/eng/index.do), PMDA (https://www.pmda.go.jp/english/), NMPA (https://english.nmpa.gov.cn/) for key words of guidance (or guideline) and combined (or combination) and/or vaccines.

Then, these published regulatory documents were reviewed by two assessors separately, and the sources were selected based on their relevance and coverage of the DTPCV and related components. Additionally, considering the lack of marketing higher-valent combined vaccines in China, NMPA draft guideline, which may partially represent the regulatory considerations, were also included in the documents for comparative analysis.

### 2.3 The descriptive and comparative design

The descriptive design was carried out from extracted subjects and related key contents from above documents between different regulatory agencies. We also analyzed the principle issues by way of comparative design, to identify major considerations in common and specific regulatory requirements.

### 2.4 The empirical study and strategic analysis

Based on the comprehensive characterization and comparison of regulatory requirements from agencies, we conducted an empirical comparative analysis to identify cases of Hep- containing DTPCVs handled by different NRAs (such as FDA, EMA, NMPA and MFDS). Using the DTPCV products (hexavalent vaccines) identified in the previous step, we searched the NRA websites for clinical reviews (or assessment reports) and other related regulatory documents, and reviewed these documents to extract relevant information, including the characteristics of clinical trials, immunization schedules, and modalities of cooperative development. Finally, the regulatory strategies employed by different agencies were analyzed and assessed using confirmed registration cases and their characteristics.

## 3 Results

### 3.1 Global guidelines for combination vaccines and representative DTPCVs

**Fig. 1:**
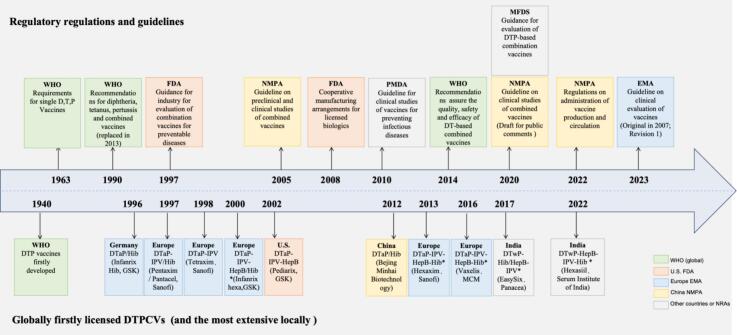
Regulatory and licensing milestones of diphtheria-tetanus-pertussis-containing vaccines (DTPCV). *Representing the major marketed hexavalent vaccines worldwide.Hexaxim and HexaSIIL were included in the WHO list of prequalified vaccines in 2014 and 2023 respectively. Besides, Pantaxim was licensed in China in 2011 and Vaxelis was licensed in U.S. in 2018. “New Meilianjitai” (DTaP/Hib) was from Beijing Minhai Biotechnology (https://www.biominhai.com/product/11.html).

By searching the regulatory documents issued by the WHO and the NRAs of the United States (FDA), European Union (EMA), South Korea (MFDS), Japan (PMDA) and China (NMPA) regarding the development and registration of combination vaccines, we identified ten regulatory documents that are now mainly in effect, and we listed them according to the issuer and issue time (Fig. 1 and Supplementary Table 1).

The guidelines for the development and registration of DTPCVs first originated from the WHO's technical requirements for D, T, and P individual vaccines, and formed the world's first DTP-based combination vaccine recommendation document in 1990. Subsequently, in 2014, WHO published the latest version “Recommendations to assure the quality, safety and efficacy of DT-based combined vaccines” (abbreviated as WHO Recommendations of DT-based vaccines) [14], which searve as the current guideline for DTPCVs.

The FDA issued the first combination vaccine guidance in 1997, which was named”Guidance for industry for the evaluation of combination vaccines for preventable diseases: production, testing and clinical studies” (abbreviated as FDA guidance of combination vaccines) [19], and published the guidance for “Cooperative manufacturing arrangements for licensed biologics” (FDA guidance of cooperative manufacturing) in 2008 [17], which serves as a technical guideline for the collaborative development of DTPCVs by different manufacturers. The EMA described possible scenarios in clinical trials of combination vaccines in its latest guidance namely “Guideline on clinical evaluation of vaccines” (abbreviated as EMA clinical guidance of vaccines), which came into effect in 2023 [20].

The MFDS published “Guidance for evaluation of DTP-based combination vaccines” (MFDS guidance for combination vaccines) [21] in 2020 to guide the development of combination vaccines in Korea, and the guidance took a very similar structure to the WHO guideline in 2014. The PMDA does not have a specific guideline for combination vaccines, but one section in its “Guideline for Clinical Studies of Vaccines for Preventing Infectious Diseases” (PMDA guideline for vaccine clinical studies), published in 2010, describe PMDA's considerations for combination vaccine development [22]. In China, the NMPA issued “Guideline for preclinical and clinical studies for combination vaccines” (NMPA 2005 guideline for combination vaccine) in 2005. However, later in 2020, the NMPA issued another draft guideline for public comments namely “Guideline on clinical studies of combined vaccines” (abbreviated as NMPA clinical guidance of combined vaccines) [23], and published “Regulations on administration of vaccine production and circulation” (abbreviated as NMPA manufacturing regulations of vaccines) [24] in 2022, which allowed the development and production of combination vaccines between pharmaceutical companies (Supplementary Table 1).

Currently,DTPCV products are predominately manufactured by three multinational vaccine companies, namely GSK, Sanofi and MCM (a joint venture between Sanofi and Merck); these three companies released their highest-valent hexavalent vaccines in 2000, 2013 and 2016 respectively. Their products, namely Infanrix-Hexa, Hexaxim and Vaxelis, have been widely licensed by the United States (excluding Infanrix-Hexa, since GSK does not market Infanrix-Hexa in the United States), the European Union, South Korea, Malaysia, and other developed and developing countries and regions. These DTPCVs are acellular pertussis (aP)-based vaccines (DTaP-IPV-HBV-Hib). Meanwhile, Indian companies have also marketed two hexavalent whole-cell pertussis (wP)-based vaccines (DTwP-IPV-HBV-Hib) in 2017 and 2022, respectively^25^(Fig. 1).

### 3.2 Regulatory considerations in registration of combination vaccines

#### 3.2.1 Feasibility of cooperative development and flexible multisite manufacturing

For many vaccines, manufacturing of bulk (drug substances) and formulations, filling, packaging and labeling are performed at different sites to manufacture the drug product. Meanwhile, the component antigens have to be produced at defined, good manufacturing practices (GMP)-compliant manufacturing; these antigens have to be adequately characterized, specific from batch to batch, and produced in NRA-approved facilities. A combination vaccine is not a random mixture of vaccines, and its development and manufacturing processes are highly complex and specialized. Globally, companies with experience in single vaccines can cooperate to develop combination vaccines, and manufacturing of bulk (drug substance) and formulations can be performed at different sites, which can reduce the cost of development and promote the uptake of combination vaccines. In 2008, the FDA guidelines on cooperative manufacturing described the most common cooperative modalities for supporting manufacturing flexibility, namely, divided manufacturing, contract manufacturing and shared manufacturing; these modalities commonly involved the production of a drug substance (DS) by one manufacturer and a drug product (DP) by another. This modality also allowed cross-company collaborations between biologics license application (BLA) holders of single vaccines, primarily through the use of the collaborating company's site for manufacturing components as intermediate (for further manufacturing) and shipping them to the formulation site for creating the final DP. In addition, the FDA guidance of combination vaccines also points to the concept of joint ventures, and the specific requirements for such ventures are referenced in the FDA guidance for cooperative manufacturing published in 2008. In 2022, following the regulations on the administration of vaccine production and circulation in China, divided manufacturing of DS and DP was allowed for combination vaccines, when the existing vaccine production capacity was unable to meet the demand and was subject to NMPA approval (Table 1).

**Table 1:** Regulatory considerations in the registration of new combined vaccines.

Topics	Subjects	Agencies	Key regulatory considerations or recommendations
Immunization schedule issues between combined and single (individual) vaccines	Convergence of immunization schedules	WHO(2014)	• Allow evaluation in the target population to provide data. • No restriction on schedule design.
		FDA(1997)	• When different schedules from approved component vaccine and new combination vaccine, submitting data showing adequacy of the proposed schedule. • Changes in doses and schedule should be supported by clinical data.
		MFDS (2020)	• No restriction on schedule design.
		PMDA (2010)	• No restriction on schedule design.
		NMPA (2005)	• No restriction on schedule design.
		NMPA (Draft in 2020)	• Only single (individual) vaccine with same or similar schedules of NIP program has the possibility of combination. • Only after validating the reasonableness of schedule changes in NIP program for a single vaccine; • In absence of data support, not recommended to change the current immunization schedules of a NIP single vaccine for developing a combined vaccine.
Clinical evaluation scenarios	Comparator vaccine and clinical trial design	WHO(2014)	• Randomized and controlled trial; • control group receiving one or more approved vaccines that contain the antigens in the new combination.
		FDA(1997)	• Randomized and controlled trial; • Control group receiving separate, simultaneously administered components contained in the combination vaccine. (If vaccine components already included in a licensed combination, the current licensed formulation could be used in control group for comparisons)
		NMPA (Draft in 2020)	• A step-by-step evaluation of the inconsistent immunization schedules • Studies on schedule changes of individual vaccines as a basis for combined vaccines development. • Combined vaccines compared with individual vaccines with changed schedule. (When individual vaccines involving NIP, changes should be carefully considered, and sufficient data should be accumulated.)
	The sources effect of individual components	WHO(2014)	• The source of components is not expected to influence the overall design of clinical evaluations.
		NMPA (Draft in 2020)	• Components of single and combined vaccine are the same (preferably the same holder), the safety and efficacy of this component can be scientific evaluated • If not the same (or even not the same holder), the scientific evaluation of the vaccines faces challenges.
	Single and combination vaccines together in one schedule	WHO(2013)	• As birth dose is never in combination with other vaccines, the comparison could be made following the first dose, which is particularly important in the prevention of maternal transmission of hepatitis B virus.
Feasibility of cooperative development in combined vaccines	Divided manufacturing of vaccines	FDA(1997)	• When two or more manufacturers wish to cooperate in the production of a combination vaccine, they should consult FDA's Policy Statement on “Manufacturing Arrangements for Licensed Biologics”
		FDA(2008)	• Divided manufacturing is an arrangement in which two or more manufacturers, each registered with FDA in accordance with 21 CFR Parts 207, 607, or 807 as applicable, and licensed to manufacture a specific biological product in its entirety, participate jointly in the manufacture of that product.
			• A common shared manufacturing agreement is one in which one manufacturer is responsible for an intermediate product and another for the final product. An application/supplement for an intermediate product licensed “for further manufacturing use” must include, in addition to other information in a BLA, the criteria used to determine lot-by-lot acceptability of the product (21 CFR 601.2) included
		NMPA (2022)	• When production of polyvalent and combined vaccines, the scope of production should be all processes. If necessary, the divided manufacturing of vaccine bulks and formulation process should be approved by the NMPA. • Divided manufacturing of DS and DP is applicable for combined vaccines, when the existing vaccine production capacity is unable to meet the demand and subjected to NMPA approval.

#### 3.2.2 Alignment of immunization schedules for combination vaccines

Public health bodies, determine immunization schedules on the basis of epidemiology, schooling age, NIPs, the available licensed vaccines, and, at the global level, the WHO Expanded Progamme on Immunization (EPI). National immunization schedule have to be regularly updated to resolve schedule differences between monovalent and combination vaccines based on disease burden, clinical effectiveness, international experience, and research [11]. From the perspective of NRAs, most regulatory guidelines across countries are flexible and generally indicate that dose schedules must be supported by clinical data. However, long-term differences in immunization schedules between the vaccines to be combined may create a dilemma in the development of combination vaccines, in such situations, awareness of local considerations and cooperation between the NRA and public health bodies is important to solve potential conflicts in immunization schedules.

Globally, most guidelines allow clinical trials of vaccines with inconsistent immunization schedules to collect data for validation purposes. For example, the WHO recommendations for DT-based vaccines state, “ Whenever possible, the combined vaccine should be evaluated in the target population following the intended schedule.” The FDA guidance for combination vaccines also implicitly states, “If the combination vaccine is to be given on a different schedule from that of any previously approved component, data showing adequacy of the proposed schedule should be submitted,”“and”Changes in dose or schedule of doses for individual components should be supported by the clinical data.” Furthermore, the FDA guidance generally advises clinical studies to demonstrate the safety, immunogenicity and efficacy of combination vaccines, including comparisons of the separate but simultaneously administered individual vaccines with the combination, and states “the data required to support each indication will be the same as for single component vaccines”, However, it dose not specify the timing to submit such data. Nevertheless, the MFDS guidance for combination vaccines, the PMDA guideline for vaccine clinical studies, and the NMPA 2005 guideline for combination vaccines, all of which discuss the development of combination vaccines, do not mention of such circumstances where immunization schedules between vaccines to be combined are different. The absence of these statements indicates that the choice of the vaccines to be combined and the design of the new schedule are not restricted.

In the NMPA clinical guidance for combination vaccines (drafted in 2020), one section discusses scenarios in which the new schedule of the combination vaccine is different from that of some of its component vaccines. For vaccines already in the NIP, the NIP is considered as the gold standard for the new combination vaccine's schedule. When the schedule of one component of the new combination vaccine is different from that in the NIP, “One should first verify the schedule change of that component standalone vaccine, before considering the feasibility of combination vaccine development.”(Table 1).

#### 3.2.3 Clinical evaluation scenarios for registration of combination vaccines

Globally, regulatory guidelines have provided comprehensive recommendations for the clinical evaluation of combination vaccines. The WHO recommendations for DT-based vaccines [15] five scenarios for new combination vaccines: adding a new antigen, replacing one antigen with another antigen for the same indication, removing an antigen, making a significant change to the manufacturing process or formulation, or a completely new manufacturer. The same logic is reflected in the MFDS guidelines for combination vaccines.

The WHO recommendations for DT-based vaccines and the FDA guidance for combination vaccines state that, “the safety and immunogenicity of a new combination should be compared in a randomized, controlled trial with the safety and immunogenicity of one or more approved vaccines that contain the antigens in the new combination,” and “the selection of the comparator vaccine should take into account the study population, the proposed immunization schedule, the total antigen composition of the candidate vaccine, and previous clinical experience with the comparator vaccine.” In addtion, an extra recommended trial design may be used when justified and approved by the NRA.”

In addition, new combination vaccine registration will also face clinical evaluation issues such as the source effect of individual components, and the use of single and combination vaccines together in one schedule.

For example, for clinical evaluation of HepB-containing combination vaccines, the WHO recommendations for HepB vaccines states [25] that “ As the birth dose is never in combination with other vaccines, the comparison could be made following the first dose, which is particularly important in the prevention of maternal transmission of hepatitis B virus.” Therefore, the trial design for clinical evaluation of the HepB-containing DTPCVs can be based on the first dose of the HepB single vaccine, followed by other equivalent HepB-containing DTPCVs. At the same time, the WHO recommendations for DT-based vaccines [15] do not consider the impact of the source of the components on the overall clinical evaluation design, such as when a manufacturer uses one or more components purchased from another manufacturer to formulate the final vaccine.

However, in the NMPA 2020 clinical guidance for combination vaccines, the source of the component is considered a major issue influencing the evaluation of clinical trials. The guidance states, “If the standalone vaccine's component is different to the corresponding component in the combination vaccine (or even not the same holder), in the case where two vaccines completed the full primary immunization schedule together, then it is very challenging to evaluate the safety and efficacy of any of these vaccines.” For example, the birth dose of the HepB vaccine was considered to interfere with the evaluation of subsequently administered HepB-containing combination vaccines (Table 1).

### 3.3 Empirical study of HepB-containing DTPCVs

#### 3.3.1 Cooperative development and flexible manufacturing strategies

Flexible multi-site manufacturing is a common strategy employed by global vaccine companies (MCM, GlaxoSmithKline, Sanofi, etc.), that can improve R&D and manufacturing efficiency through extensive production networks. In practice, vaccine production involves three principal stages: (1) manufacture of active ingredients or DSs, (2) transformation of the ingredients into DPs or vaccines, and (3) final packaging (summarized from the Sanofi website, https://www.sanofi.com/en/your-health/vaccines/a). For DTPCVs, the individual bulks of the combination vaccine components and the formulations can be produced at a number of sites with a divided manufacturing modality.

Infanrix-hexa,which was first licensed in 2000 by the EMA, was considered a new combination of approved antigens, because all component vaccines had been licensed by the EMA before licensure of the hexavalent vaccine, and all components were manufactured by one applicant (GSK) (e.g., Infanrix HepB: D, T, aP, and HBV; Infanrix IPV: D, T, aP, and IPV; Hiberix: Hib). Based on the information disclosed in the assessment reports, the Infanrix-Hexa product was manufactured at two sites, Germany Chiron-Behring site (DS) and Belgium SB Biologicals site (DS and DP). Both sites had validated manufacturing licenses demonstrating compliance with GMP requirements (Table 2).

**Table 2:** Divided manufacturing and manufacturing sites for hexavalent vaccines.

Divided manufacturing	Bulk components	Manufacturing sites	NRA
Infanrix hexa (lyophilised reconstitution), GSK, licensed in 2000 by EMA			
Intermediate products (DS)	DT Bulk	GSK (Chiron-Behring, Germany site)	EMA
	T Bulk for Hib component		
	aP Bulk	GSK (SB Biological, Belgium)	EMA
	HBV Bulk		EMA
	IPV Bulk		EMA
	Hib component Bulk		EMA
Finished products (DP)	DTPa-HBV-IPV/Hib	GSK (SB Biological, Belgium)	EMA
Hexaxim (fully liquid), Sanofi, licensed in 2013 by EMA			
Intermediate products (DS)	HBV Bulk	Sanofi Pasteur (Pilar sites)	EMA
	Hib Bulk	Sanofi Pasteur (France sites)	EMA
	IPV Bulk		EMA
	DTaP Bulk		EMA
Finished products (DP)	DTPa-HBV-IPV-Hib	Sanofi Pasteur (France sites)	EMA
Vaxelis (fully liquid), MCM, licensed in 2016 and 2018 by EMA and FDA			
Intermediate products (DS)	Hib Bulk (For Further Manufacturing Use)	Merck Sharp & Dohme (U.S. sites)	FDA & EMA
	HBV Bulk (For Further Manufacturing Use)		
	IPV Bulk	Sanofi Pasteur (France sites)	EMA, (b)(4) to FDA
	DTaP Bulk	Sanofi Pasteur (Canada sites)	FDA & EMA
Finished products (DP)	DTaP-HBV-IPV-Hib	Sanofi Pasteur (U.S. sites)	EMA
		Sanofi Pasteur (Canada sites)	FDA

Abbreviations:Drug substance, DS; Haemophilus b Conjugate, Hib; Inactivated Poliovirus, IPV; Diphtheria and Tetanus Toxoids, acellular Pertussis, DTaP; Hepatitis B Vaccine (Recombinant) or HBsAg, HBV; The vaccine concentrates of components in Infanrix-Hexa is also called bulk. The drug product (DP) is the finished products of DS, followed by formulation, filling, labeling and packaging. Source of data derived from reviews and assessment reports of hexavalent vaccine products.

Similarly, Hexaxim, another hexavalent vaccine, licensed in 2013 by the EMA, was also cooperatively produced at Argentina Pilar and France sites. Based on the information disclosed in the assessment reports, all components were licensed and manufactured by one applicant (Sanofi Pasteur), but only the HBV bulk was produced at the Pilar site (now belonging to Sanofi Pasteur), which is considered an investigational HepB vaccine with data from two phase 3 clinical studies in Argentina and Uruguay [26]. Furthermore, the EMA declared that studies of the candidate combination vaccine had already been considered, even if no study reports were available for these two studies of the HepB vaccine. It indicated that combination vaccines can incorporate investigational components (e.g., HBV DS) if validated correlates of protection (CoPs) exist (e.g., anti-HBs ≥ 10 mIU/mL; D/*T* ≥ 0.1/0.01 IU/mL; Hib ≥ 0.15 μg/mL; IPV ≥ 1:8 titers); this approach simplified regulatory review, as demonstrated in the case of Hexaxim [27] (Table 2).

To support the cooperative development of the hexavalent vaccine, Merck and Sanofi Pasteur formed a joint venture with the MCM Vaccine Company in 1991, and the hexavalent vaccine Vaxelis (MCM) was licensed in 2016 by the EMA and in 2018 by the FDA. Vaxelis combines the licensed vaccine components of the two companies, with Diphtheria, Tetanus, Pertussis and Polio antigens provided by Sanofi Pasteur, and Hib and Hepatitis B antigens provided by Merck. In the United States, as recommended by the FDA guidance (2008), Merck submitted two separate Further Manufacturing Use (FFMU) BLAs for HepB surface antigen (HBsAg; STN 125581) and *Haemophilus influenzae* type b (PRP-OMPC, STN 125580), and provided chemistry, manufacturing and control (CMC) information specific to the two intermediate antigens and their use in Vaxelis production. .

Vaxelis is also cooperatively produced at multiple sites. For Vaxelis licensed in the United States, intermediates (bulks) produced at different manufacturing sites (Merck and Sanofi Pasteur) are shipped to Sanofi's manufacturing sites for final products, and the formulation, filling, and packaging were completed by Sanofi Pasteur Limited in Ontario, Canada. Similarly, for Vaxelis licensed in Europe, the intermediate products are manufactured at the same manufacturing site as in the United States, but the filling was performed by Sanofi Pasteur Inc. in Swiftwater, USA. (Table 2).

#### 3.3.2 Supporting evidence for aligning immunization schedules

Inconsistent schedules may pose challenges in the and registration of the HepB containing DTPCVs. For example, the schedule of the conmmonly used HepB-containing hexavalent vaccine (2–4-6 months or 6–10-14 weeks, etc., as recommended by the WHO) is inconsistent with that of the Hep B vaccine in the current immunization program (0–1-6 months) in some countries. A WHO position paper (2017) [28] noted the need for flexibility in the HepB vaccine immunization schedule and recommended that after the birth dose, the HepB vaccine (mono- or combined) should be administered simultaneously with the DTP vaccine. Currently, a delay in the second dose of the HepB vaccine from 1-month to 2-months of age for infants of HBsAg-negative mothers has been widely accepted. Based on the epidemiology of HepB, regulatory agencies have allowed the development of Hep B-containing hexavalent vaccines using different regulatory strategies. In summary, we classified the regulatory strategies into two patterns: prospective and accelerated. The former refers to the strategy employed by those who first developed HepB-containing DTPCVs in the world, for these scenarios,t vaccine development was innovative and prospective, without scheduling issues. The latter refers to the strategy employed by those who developed HepB-containing DTPCVs subsequently, since they may have had to address the convergence issues caused by potential schedule differences.

The prospective regulatory strategy in the United States.

A typical case of a prospective strategy can be illustrated by the development history of DTPCVs in the United States. As a country with a low prevalence of the HepB virus, the HepB vaccine immunization schedules evolved over a 10-year period from 1995 to 2006. After 2006, the immunization schedules of single HepB vaccines did not differ from those of hepatitis B-containing hexavalent vaccines. Overall, the combination vaccines and the corresponding single vaccines were programmed to be injected at the same time points, enabling the transition from a single vaccine to a component of a hexavalent vaccine.

In 2018, without immunization schedules issues, a hexavalent vaccine (DTaP-IPV-Hib-HepB) was licensed in the United States. by the FDA. In 2018, the United States Advisory Committee on Immunization Practices (ACIP) recommended an immunization schedule in which infants receive the first dose of the HepB vaccine at birth and the second dose in the form of hexavalent vaccine at 2 months of age.

The accelerated regulatory strategy in some Asian countries.

As latecomers in the development of HepB containing hexavalent vaccines, some Asian countries with an intermediate prevalence of HepB, such as Korea and Malaysia, adopted an accelerated strategy. In these countries, the current HepB vaccine immunization schedule was 0–1-6 months, which usually did not match the first dose of the common schedule (2-month of age) of HepB-containing DTPCVs. Accordingly, the performances of DTPCV products with new schedules and related component vaccines with the original schedules were evaluated simultaneously in the same clinical trial, which further accelerated the licensing of DTPCVs.

**Fig. 2:**
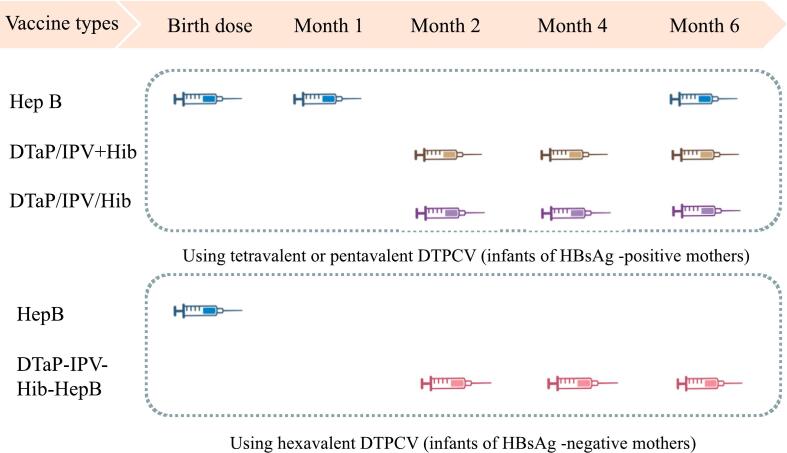
The immunization schedules of HepB-containing vaccines in Korea**.**

Note: In Korea, for children of hepatitis B-positive mothers, the hepatitis B immunization strategy is recommended to vaccinate at 0–1-6 months of age to complete the vaccine series after receipt of the hepatitis B immune globulin (HBIG).

In 2020, based on WHO recommendations, the MFDS approved the hexavalent vaccine (DTaP-IPV-Hib-HepB). Thus, South Korea had independent immunization schedules for HepB-containing hexavalent vaccines, In thi system, infants were classified as born from HBsAg-positive- and negtive-mothers to account for possible infection risks, and the Korean Pediatric Association recommends the use of the hexavalent vaccine in neonates of HepB-negative mothers with a 2–4-6 months age schedule after birth dose [29] **(**Fig. 2**)**.

#### 3.3.3 Clinical designs in considerations of epidemiology and component sources

Considerations of HepB risk control and component sources in the United States.

Currently, only one hexavalent vaccine, namely Vaxelis, is licensed by the FDA (in 2018). In the phase III registered clinical trial V419–005 (PR505) conducted in the United States (low prevalence of hepatitis B), subjects who had already been vaccinated with different brands (component sources) of the HepB vaccine at birth were enrolled for clinical trials. The experimental group was vaccinated with the HepB-containing hexavalent vaccine Vaxelis (2–4-6 months of age), whereas the control group was vaccinated with the pentavalent vaccine Pentacel (2–4-6 months of age) and the single HepB vaccine RECOMBIVAX HB (2–6 months of age). When the clinical trial was completed, the investigators also conducted subgroup analyses among subjects who received HepB vaccine birth dose of different brands, and further analyses confirmed that the branded of vaccine administerd in the birth did not influence immune responses.

In addition, no restrictions related to maternal HBsAg results were forced for enrollment in the trial, and all infants were vaccinated with a single hepatitis B vaccine at birth. In accordance with the vaccines recommended by the United States NIP, all infants were simultaneously immunized with the Rotavirus Vaccine RotaTeq (2–4-6 months) and the Pneumococcal Vaccine Prevnar 13 (2–4–6-15 months) (Table 3).

**Table 3:** Regulatory strategies and considerations in registration studies of the hepatitis B containing DTPCVs.

Regulatory strategies	Representative countries	HepB prevalence	Enrollment control	Practices in clinical studies
Prospective strategy	U.S.	low	No restriction of results for HBsAg test	• Enrollment subjects vaccinated with different brands of hepatitis B vaccine for birth dose. • Conducting subgroup analyses of the hepatitis B immune responses when clinical trial completed
Accelerated strategy	Korea	Middle to low	A stratified enrollment for infants of HBsAg-negative mothers	• Enrollment subjects vaccinated with a common brand (used in NIP) of hepatitis B vaccine for birth dose.

Considerations for HepB risk control and component sources in Korea.

Another case focused on the hexavalent vaccine Hexaxim, which was licensed by the Korean Food and Drug Safety Administration (MFDS) in 2020. In Korea (intermediate prevalence of hepatitis B), a local phase III clinical registration study (A3L31) of Hexaxim was conducted in 2014, and constrained enrollment was performed. The MFDS only allowed the enrollment of 1-month-old infants of HBsAg-negative mothers (>300 subjects), who had received birth doses of locally produced hepatitis B vaccine (Euvax B, LG) widely used in the Korean National Immunization Program. At the age of 2–4-6 months, subjects in the experimental group were vaccinated with Hexaxim, and the subjects in the control group were vaccinated with the pentavalent vaccine without HepB and the HepB monovalent vaccine from the current standard regimen in Korea. The study design allowed the evaluation of Hexaxim, especially for the hepatitis B component, after the HepB birth dose from a different brand [30]. Overall, in the stratified enrollment in clinical trials, only infants from HBsAg-negtive mothers were grouped and vaccinated in accordance with the original and new immunization schedules (Table 3).

## 4 Discussion

### 4.1 Flexible and cooperative strategy to accelerate development in future

In both in guideline requirements and case studies, cooperative development and flexible multi-site manufacturing of combination vaccines were very common. Individual bulks manufactured at different sites can contribute to the production capacity. In countries where no hexavalent vaccines are available, this approach represents an efficient strategy is to accelerate development when individual vaccines are licensed locally.

Considering companies in China as an example, in the current situations, production of combination vaccines can be performed cooperation among subsidiaries within the same parent company However, China does not yet have practical experience in the commercial divided manufacturing of combination vaccines, probably because penta- and hexaDTPCV products have not yet received local licenses (Supplementary Table 2) [31]. With advancements in divided manufacturing, more locally produced high-valence combination vaccines are expected to become available in China.

### 4.2 Possible actions to harmonize the mismatched schedule

For schedule harmonization, adequate data should be submitted to demonstrate similar performances between vaccinations according to the proposed and existing schedules. When combination vaccines are evaluated in some countries, especially for the HepB containing DTPCVs, caution is requied because of the existing NIP and epidemiological considerations.

In fact, the United States and most countries of the European Union are low-endemic areas for the hepatitis B virus, while middle-to-high endemic areas are in China, South Korea, Malaysia and some Asian countries [32]. To better control epidemics, the HepB vaccine is usually administered in a schedule of 0–1-6 months of age [33]. Additionally, in China, the NMPA clinical guidance for vaccines [23] included special considerations stating that, “If a combination vaccine is developed that is inconsistent with the schedules of NIP program, it may reflect a mismatch with the disease prevention and control needs”. Since the hepatitis B vaccine is an NIP vaccine, adjustment of the immunization program for schedule adaptation may be difficult. Conversely, making NIP compliance a prerequisite for combination vaccine development may be unnecessary, since this requirement restricts the acceptance of clinical trials of hepatitis B-containing DTPCVs.

Recent studies have yielded extensive cumulative evidences supporting a second dose of the HepB vaccine at 2-months of age in infants of HBsAg-negative women. A Korean study [34] showed that even among infants of HBsAg-positive mothers who received the first dose of the HepB vaccine and immunoglobulin in a timely manner, administering the second dose of the HepB vaccine at 2-months of age did not increase the risk of hepatitis B infection in infants, in comparison with administering the second dose at 1- month age. In China, delays in second dose of the HepB vaccine are not uncommon, although an immunization schedule that delays the second dose until 2-months of age has not been recommended. In China, the percentage of infants who received the second dose of HepB vaccine at 2-months of age and later during the period 2019–2021 reached 10.59 % [35]. Meanwhile, immunization programs with a birth dose of the HepB vaccine and subsequent doses of HepB -ontaining DTPCVs at 2–4-6 months, have been applied globally in infants, irrespective of the mothors' HBsAg-positive status^29^.

Thus, based on considerations of risk control and schedule flexibility, a stratified immunization schedule for infants with mothers who are HBsAg-positive and HBsAg-negative should be established and clinical trials should be conducted to collect evidence, especially in countries where HepB is still endemic. In this regard, however, the prevention of hepatitis B mother-to-child-transmission (PMTCT) strategy has been shown to be effective. Following the accumulation of epidemiological data, these changes may be permitted in some low-risk populations, which will require more effective communication and coordination between health, pharmaceutical regulation, and disease control regulators and policy makers. Countries with less successful PMTCT implementation may be more cautious when adopting this strategy.

### 4.3 Clinical strategies for evaluation of combination vaccines with inconsistent schedules or different component sources

For clinical evaluation of inconsistencies between immunization schedules of combination and component vaccines, the NMPA clinical guidance of vaccines (Draft for Public Comments in 2020)included special considerations stating that “A step-by-step evaluation of inconsistent immunization schedules is needed, …… it is recommended that studies on schedule changes of individual vaccines are carried out as a basis for combined vaccines development, and that the clinical study of combined vaccines should be compared with that of individual vaccines after schedule changes”. The regulatory requirements have developed a stepwise strategy for the transition from individual to combination vaccines, and maintenance of the same sources (the same holders) of components has been emphasized to clearly define the parties responsible for post-marketing safety issues. These considerations will ensure greater caution, especially for components of NIP vaccines.

Globally, clinical experience and registration strategies for combination vaccines have adopted optimized protocols to control the potential risks. In the United States, an iterative strategy was illustrated based on the combination of licensed single vaccines with the same or similar schedules. When faced with inconsistencies in the immunization schedule between the hexavalent vaccine and HepB single vaccine, Korean regulators allowed the control of the enrollment conditions in clinical studies to decrease the known risks, and approves the conduct of a clinical trial to accumulate relevant data locally. Meanwhile, simultaneous evaluation in one study allowed schedule changes and assessment of the performances of combination vaccines in a small group of low-risk subjects. Finally, approval of the hexavalent vaccine was supported by global clinical data and sufficient local data. Additionally, retrospective subgroup analyses were conducted to evaluate the effectiveness of blending HepB monovalent vaccine of different brands, and provide more evidence of interchangeability for subsequent market use.

### 4.4 Limitations

Overall, the global regulatory landscape of combination vaccines was illustrated, with the aim of informing a broader audience of R&D and regulatory agencies about the underlying registration strategies and development gaps. However, the causes of delays in the development of combination vaccines are complex and multi-factorial, and include pharmaceutical, clinical, manufacturing, regulatory, public health and marketing issues. In this paper, we mainly shed light on the registration strategies of combination vaccines from a regulatory perspective, and further focused on key registration-related clinical considerations of DTPCVs, but not on formulation, preclinical issues of adjuvants, or compatibility of components. However, limitations still exist in that not all types of combination vaccines, other than DTPCVs, are outlined in this paper. Furthermore, the regulatory guidelines may not have been exhaustively reviewed because some regulatory documents may have been inaccessible owing to a lack of regulatory transparency. A descriptive and comparative design aligned with an empirical study may not be strongly supportive because of the lack of quantitative data analysis. In the future, the evidence for registration of DTPCVs will continue to evolve as scientific knowledge and regulatory experience increase, and will help optimize regulatory studies of DTPCVs registration.

## 5 Conclusions

DTPCVs demonstrated the highest complexity among in combination vaccines, potentially causing certain issues, especially for HepB-containing combination vaccines. In this study, we described and compared key considerations in combination vaccine registration from the regulatory documents of the WHO and national and regional agencies. We then analyzed empirical cases that reflect prospective or accelerated strategies resulting from different epidemiological and regulatory requirements. Finally, we proposed practical approaches to balance the regulatory rigor and developmental acceleration of DTPCVs. Overall, these proposals should support an understanding of regulatory registration in the face of the common DTPCV development challenges.

## Authors' contribution

H.X.C. drafted the manuscript, P.Y.W. edited a summary of the legal and regulatory considerations, L.Z.Y. added the timeline of DTPCVs development, H.S.T. commented and assisted in manuscript refinement, and Y.Y. supervised this paper. All authors have read and agreed to the published version of the manuscript.

## CRediT authorship contribution statement

**Xiangchuan He:** Writing – original draft. **Yiwen Pu:** Validation. **Zeyu Li:** Visualization. **Shitong Huan:** Supervision. **Yue Yang:** Supervision.

## Funding

This review was supported by the Bill and Melinda Gates Foundation grant [grant no. INV-046112].

## Declaration of competing interest

The authors declare that they have no known competing financial interests or personal relationships that could have appeared to influence the work reported in this paper.

## Data Availability

Administrative data are used in compliance with local regulatory and legal frameworks that govern data use.

## Appendix A Supplementary data

Supplementary material Image 1

## References

[1] Lindstrand A., Cherian T., Chang-Blanc D., Feikin D., O’Brien K.L. (2021). The world of immunization: achievements, challenges, and strategic vision for the next decade. J Infect Dis.

[2] Taychakhoonavudh S. (2020).

[3] Maman K., Zöllner Y., Greco D., Duru G., Sendyona S., Remy V. (2015). The value of childhood combination vaccines: from beliefs to evidence. Hum Vaccin Immunother.

[4] Kurosky S.K., Davis K.L., Galindo C.M. (2017). Effect of combination vaccines on hepatitis B vaccine compliance in children in the United States. Pediatr Infect Dis J.

[5] Obando-Pacheco P., Rivero-Calle I., Gómez-Rial J., Rodríguez-Tenreiro Sánchez C., Martinón-Torres F. (2018). New perspectives for hexavalent vaccines. Vaccine.

[6] Dakin A., Borrow R., Arkwright P.D. (2023). A review of the DTaP-IPV-HB-PRP-T hexavalent vaccine in pediatric patients. Expert Rev Vaccines.

[7] Syed Y.Y. (2019). DTaP-IPV-HepB-Hib vaccine (Hexyon®): An updated review of its use in primary and booster vaccination. Pediatr Drugs.

[8] Skibinski D.A., Baudner B.C., Singh M., O’Hagan, D. T. (2011). Combination vaccines. J Global Infect Dis.

[9] Boisnard F., Manson C., Serradell L., Macina D. (2025). DTaP-IPV-HB-Hib vaccine (Hexaxim): an update 10 years after first licensure. Expert Rev Vaccines.

[10] https://immunizationdata.who.int/listing.html?topic=vaccine-schedule&location=.

[11] Li J., Chen S., Asturias E., Tang S., Cui F. (2024). Promoting higher-valent pediatric combination vaccines in China: challenges and recommendations for action. Infect Dis Poverty.

[12] Zheng Y., Rodewald L., Yang J., Qin Y., Pang M., Feng L. (2018). The landscape of vaccines in China: history, classification, supply, and Price. BMC Infect Dis.

[13] Tafreshi S.-Y.H. (2020). Efficacy, safety, and formulation issues of the combined vaccines. Expert Rev Vaccines.

[14] http://www.who.int/biologicals/vaccines/Combined_Vaccines_TRS_980_Annex_6.pdf,%202012.

[15] Vidor E., Soubeyrand B. (2016). Manufacturing DTaP-based combination vaccines: industrial challenges around essential public health tools. Expert Rev Vaccines.

[16] Martinón-Torres F., Vesikari T., Van Damme P. (2021). Pediatric vaccines and vaccinations.

[17] http://www.fda.gov/BiologicsBloodVaccines/GuidanceComplianceRegulatoryInformation/Guidances/General/ucm069883.htm.

[18] Decker M.D. (2001). Principles of pediatric combination vaccines and practical issues related to use in clinical practice. Pediatr Infect Dis J.

[19] https://www.fda.gov/regulatory-information/search-fda-guidance-documents/guidance-industry-evaluation-combination-vaccines-preventable-diseases-production-testing-and.

[20] https://www.ema.europa.eu/en/documents/scientific-guideline/guideline-clinical-evaluation-vaccines-revision-1_en.pdf.

[21] https://www.mfds.go.kr/brd/m_1060/down.do?brd_id=data0011&seq=14779&data_tp=A&file_seq=1.

[22] Nomura Y., Noda K., Oohashi Y., Okuda S., Matsumoto J., Nakano T. (2022). Proposal for the revision of guidelines for clinical trials of vaccines to prevent infectious diseases in Japan. Vaccine.

[23] https://www.ccfdie.org/cn/yjxx/yphzp/webinfo/2020/12/1608323673536573.htm.

[24] https://english.nmpa.gov.cn/index.html.

[25] Chitkara A.J., Parikh R., Mihalyi A., Kolhapure S. (2019). Hexavalent vaccines in India: current status. Indian Pediatr.

[26] Tregnaghi M.W., Voelker R., Santos-Lima E., Zambrano B. (2010). Immunogenicity and safety of a novel yeast Hansenula polymorpha-derived recombinant hepatitis B candidate vaccine in healthy adolescents and adults aged 0-45 years. Vaccine.

[27] Plotkin S.A. (2010). Correlates of protection induced by vaccination. Clin Vaccine Immunol.

[28] World Health Organization (2019). Hepatitis B vaccines: WHO position paper, July 2017- recommendations. Vaccine.

[29] Cho H.-K., Park S.E., Kim Y.-J., Jo D.S., Kim Y.-K., Eun B.-W. (2021). Recommendation for use of diphtheria and tetanus toxoids and acellular pertussis, inactivated poliovirus, Haemophilus Influenzae type B conjugate, and hepatitis B vaccine in infants. Clinical and Experimental pediatrics.

[30] Kim Y.-K., Vidor E., Kim H.M., Shin S.M., Lee K.-Y., Cha S.-H. (2017). Immunogenicity and safety of a fully liquid DTaP-IPV-HB-PRP∼T hexavalent vaccine compared with the standard of Care in Infants in the Republic of Korea. Vaccine.

[31] https://english.nmpa.gov.cn/database.html.

[32] Razavi-Shearer D., Gamkrelidze I., Pan C., Jia J., Berg T., Gray R. (2023). Global prevalence, Cascade of care, and prophylaxis coverage of hepatitis B in 2022: a modelling study. Lancet Gastroenterol Hepatol.

[33] Hur Y.J., Choe S.-A., Choe Y.J., Paek J. (2021). Hepatitis B surface antigen and antibody positivity among women of childbearing age after three decades of universal vaccination in South Korea. Int J Infect Dis.

[34] Yang T.U., Vargas-Zambrano J.C., Park H.A., Jung C.W., Kim D., Jee Y. (2023). Effect of the interval between birth and second dose of hepatitis B vaccine on perinatal transmission of hepatitis B virus. Hum Vaccin Immunother.

[35] Zhang Xue, Huang Aodi, Li Ping, Tang Lin, Huang Lifang, An Jing (2023). Timeliness and inter-dose intervals of hepatitis B vaccine in children born between 2019 and 2021 in several areas of China. zhongguo Yimiao he Mianyi.

