# Supplementary material for: The global regulatory landscape for combined vaccines: A comparative case study of registration strategies for diphtheria-tetanus-pertussis-containing vaccines
Source: Vaccine. 2025 Apr 30;54:None. doi: 10.1016/j.vaccine.2025.127017 (PMC12132046; doi:10.1016/j.vaccine.2025.127017)
Supplement: Supplementary file 1 — Supplementary material [file mmc1.docx]

多联疫苗临床研究技术指导原则

（征求意见稿）

2020年11月

目 录

[一、概述 3](#_Toc57363281)

[（一）背景 3](#_Toc57363282)

[（二）多联疫苗定义 3](#_Toc57363283)

[（三）适用范围 4](#_Toc57363284)

[二、研发应考虑的立题基础 4](#_Toc57363285)

[（一）免疫程序的相同或相似性 5](#_Toc57363286)

[1. 相同/相似的评价标准 5](#_Toc57363287)

[2. 各情形下研究的可评价性 6](#_Toc57363288)

[（二）立题的其它考虑 8](#_Toc57363289)

[三、申报临床试验的条件 9](#_Toc57363290)

[（一）基本条件 9](#_Toc57363291)

[（二）具体条件 10](#_Toc57363292)

[1. 仅一个单苗未注册上市的 10](#_Toc57363293)

[2. 两个及以上单苗未注册上市的 11](#_Toc57363294)

[四、临床试验设计 11](#_Toc57363295)

[（一）通用考虑 11](#_Toc57363296)

[1. 总体设计 12](#_Toc57363297)

[2. 安全性研究 13](#_Toc57363298)

[3. 有效性研究 14](#_Toc57363299)

[（二）特殊考虑 16](#_Toc57363300)

[1. 仅一个单苗未注册的 16](#_Toc57363301)

[2. 两个及以上单苗未注册上市的 17](#_Toc57363302)

[3. 仅用于加强免疫 17](#_Toc57363303)

[（三）评价标准的调整 18](#_Toc57363304)

[五、上市后的变更 19](#_Toc57363305)

[参考文献 20](#_Toc57363306)

多联疫苗临床研究技术指导原则

# 一、概述

## （一）背景

联合疫苗可减少受种者，尤其是婴幼儿的接种次数，有利于提高综合接种率和促进传染防病控工作，具有较大的临床需求和公共卫生价值。世界卫生组织（WHO）一直积极倡导，我国《中国人民共和国药品管理法》、《药品注册管理法》以及《中华人民共和国疫苗管理》也均鼓励包括多联多价疫苗在内的药品创新。

目前我国已上市联合疫苗相比于欧美存在较大差距，主要表现为种类少和联合程度低。相关企业对联合疫苗的研发积极性正日益提高，但研发水平参差不齐，临床方面主要体现在研发的必要性和可行性、临床研究的整体思路及设计等方面。原国家食品药品监督管理局在借鉴美国食品药品监督管理局（U.S. FDA）相关指导原则（1997年版）的基础上，于2005年发布了《联合疫苗临床前和临床研究技术指导原则》，该指导原则主要关注于临床研究实施阶段的原则性要求。为更加适应目前的联合疫苗研发形势，同时从立项开始就做好各环节指导工作，现根据国内外联合疫苗研发及临床实践经验，结合我国疫苗产业现状和研发水平特点，制定本技术指导原则。

## （二）多联疫苗定义

根据2020年版《中华人民共和国药典》，**联合疫苗**指由两个或以上活的、灭活的病原微生物或抗原成分联合配制而成的疫苗，用于预防不同病原微生物或同一种病原微生物的不同血清型/株引起的疾病。联合疫苗包括多联疫苗和多价疫苗。**多联疫苗**用于预防不同病原微生物引起的疾病；**多价疫苗**用于预防同一种病原微生物的不同血清型/株引起的疾病。

本指导原则的相关定义基本与药典相同，其中建议采用按生物分类学的“种（Species）”进行多价疫苗与多联疫苗的区分。由同一种病原体的不同群、血清型或基因型的抗原构成的为多价疫苗，主要用于预防同一种疾病。含有不同种病原体相关抗原的为多联疫苗，用于预防多种疾病或同一种疾病；本指导原则中所称“单苗”是指每一种病原体对应的疫苗，单苗可能为单价疫苗，也可能为多价疫苗。

## （三）适用范围

本指导原则适用于多联疫苗的临床研究，主要包括研发时应考虑的立题基础、申报临床试验应具备的条件、临床试验设计的通用及特殊考虑。本指导原则将随着相关法规和标准的不断完善以及科学知识水平的提高适时更新。

对于目前在生物学上分类尚未形成国际共识的病原体开展联合疫苗研究的，为保证受试者安全和降低研发失败风险，建议参照多联疫苗进行研究。

# 二、研发应考虑的立题基础

多联苗的研发应首先从传染病预防的角度考虑其临床需求，主要为各单苗免疫程序的相同或相似性；同时从临床研究角度考虑研究结果的可评价性，综合评估联合的必要性和可行性。

## （一）免疫程序的相同或相似性

接种多联疫苗等同于同时接种多个单苗，因此具有相同或相似免疫程序的单苗才具有联合的可能性。

### 1. 相同/相似的评价标准

#### （1）国家免疫规划程序

国家免疫规划（National Immunization Program，NIP）疫苗的免疫程序（包括起始接种月/年龄、间隔、剂次等）契合了相应时间段传染病防控的实际需求，并经临床研究数据的积累最终确定，目的是在发病高峰（月龄）前建立免疫保护并有效地预防疾病。拟联合的单苗中若有NIP疫苗的，则应优先以具有统一接种程序的NIP疫苗为标准，来考虑拟与NIP联合的其他单苗的免疫程序相同或相似性。因此若多联苗研发出现与NIP程序不相符，可能反映的是与NIP所代表的疾病预防控制需求的不匹配。各国的传染病流行特点存在不同，各国的NIP程序也因此存在差异，故境外具备临床应用基础可研发的多联苗在我国的适用性需在立题时充分考虑。

另一方面，由于疾病流行特征发生改变、免疫程序不断完善以及疫苗的生产工艺不断优化，NIP也存在免疫程序改变的客观需求。应首先验证单苗NIP程序改变的合理性，在此基础上再考虑多联疫苗研发的可行性；在缺乏数据支持的情况下不建议直接改变某个NIP单苗的现行免疫程序用于多联苗的研发。

#### （2）非国家免疫规划程序

如拟联合的多联苗均为非NIP疫苗，则建议优先以其中已广泛应用且免疫程序较为统一的单苗为标准，评价其它单苗与其免疫程序的相似程度，原因同NIP疫苗。类似地，参照境外已有多联苗考虑境内研发时，也需基于疾病预防的实际需求在立题时充分评估适用性。

### 2. 各情形下研究的可评价性

基于免疫程序相同或相似的各种具体情形，结合临床研究的可评价性提出关于研发立题的考虑建议。

#### （1）基础免疫程序完全相同

免疫程序相同包括各单苗的全部免疫程序完全相同，即基础免疫以及加强免疫程序均相同；也可包括单苗仅基础免疫程序完全相同而加强免疫程序不同。两种情况下就基础免疫均可直接考虑多联苗的研发。

#### （2）基础免疫程序部分相同

如果基础免疫仅部分剂次相同，所不同程序的剂次则需以相应单苗进行接种，那么全程基础免疫后该单苗成分的安全有效性则由两种疫苗（单苗和多联苗相应成分）共同形成。这种情况下，若单苗与多联苗中的相应成分相同（最好为同一上市许可持有人的产品），则该成分安全有效性的最终评价具备一定的科学依据，多联苗也相应具有较好的研发基础。若拟接种的单苗并非多联苗中的相应的成分（甚至不为同一上市许可持有人的产品），则由两种疫苗/成分共同完成全程免疫的情况下，科学的评价其中一种疫苗的安全有效性则面临极大的挑战。同时，不管是临床研究还是工作实践中，并不推荐混合使用不同上市许可持有人的疫苗完成预防接种，这会给受种者带来更大的安全性风险。综上，为更科学地评价疫苗的安全有效性，以及更有利于产品的质量控制，落实上市许可持有人的主体责任，建议研发者在同时具备各单苗研发能力的前提下开发多联苗，并尽量保证成分的一致性。

#### （3）基础免疫程序相似

现有的免疫程序具有相似性也为多联疫苗的研发提供了一定的可行性，但并不意味着可直接研发，必须基于单苗的免疫程序经临床研究确认可变更以实现各单苗的免疫程序相同。建议先行开展单苗免疫程序变更的研究作为研发基础，多联苗临床研究阶段应与程序变更后的单苗进行比较。单苗免疫程序变更若涉及NIP疫苗的应更慎重，需积累足够的数据。

#### （4）仅用于加强免疫

类似于基础免疫程序部分相同的情况，仅基于加强免疫剂次研发的多联疫苗其加强免疫的安全有效性由基础免疫和加强免疫的不同疫苗构成，客观评价加强剂次疫苗同样面临挑战。原则上多联疫苗鼓励从基础免疫需求着手进行研发。与基础免疫各剂次间隔一般相对较短（多为1个月）不同，加强免疫一般间隔较长（多为1-1.5年后），因此在良好的试验设计基础上，仅用于加强免疫的多联苗可能具有一定可评价性，但需认识到混合使用不同疫苗的接种还是会带来安全性风险的增加。

加强免疫是作为个体建立免疫保护所必须的免疫程序之一，提醒应与复种或强化免疫等基于群体的疾病预防控制措施相区别。

## （二）立题的其它考虑

除了从临床应用的角度考虑程序的匹配性和数据的可评价性，多联苗在立题时还需从物质基础的角度进行必要的药学考虑。例如部分单苗的佐剂对其它不含佐剂单苗抗原成分的可能影响以及相应的工艺要求；多联苗较之单苗抗原含量调整的必要性和合理性；防腐剂、其它辅料/非活性成分、稳定期和有效期等的影响和考虑等。可参见已有的《联合疫苗临床前和临床研究技术指导原则》、《预防用含铝佐剂疫苗技术指导原则》或其他后续发布的指南性文件，上述情形建议在多联苗研发的立题阶段即开始考虑，必要时还需临床阶段进行验证。

# 三、申报临床试验的条件

## （一）基本条件

多联苗较之单苗成分更多，相对的安全性风险更高，在缺乏单苗安全有效性证据的情况下直接开展多联苗的临床研究对于受试者来说是不合理的；同时多联疫苗不应降低单苗单独接种的安全有效性或影响程度可接受，也需获得单苗的安全有效性数据进行比较。因此，组成多联疫苗的所有单苗（或成分）能够确认具有或能够合理预期具有安全有效性（尤其是安全性），是多联苗申报临床试验的基本条件。从实践看，境外疫苗企业现有的多联疫苗，几乎都是基于单苗已获批上市后再开展研发的。

临床试验是单苗获得安全有效性数据最为直接的方式；生产工艺相同且成熟的、已广泛应用的同类疫苗也能够一定程度上借鉴安全有效性特征。原则上鼓励完成单苗的上市注册后再行申报多联苗的临床试验。为鼓励多联苗研发，可视单苗的创新程度和安全有效性合理预期的把握度，在单苗临床研究不同阶段申报和开展多联苗的临床试验；若单苗较为成熟的可接受单苗与多联苗同期研发。

我国现有的麻腮风联合减毒活疫苗、基于共纯化技术的吸附无细胞百白破联合疫苗虽为多联疫苗，但研发、评价和实践使用均较为成熟。在与其它单苗联合进行临床试验时，可暂将其作为整体进行考虑，开展研发和临床评价工作。但当涉及物质基础的药学改变并经评估需要临床验证时，则按照三联苗进行要求。

## （二）具体条件

对于组成联苗的各单苗，最为成熟的情况为各单苗均已注册上市，且免疫程序相同或相似，在此不再赘述。以下按照一个单苗未注册上市、两个及以上单苗未注册上市等情形，分别考虑多联苗申报临床试验应具备的具体条件。

### 1. 仅一个单苗未注册上市的

以二联苗为例，当其中一个单苗为研发者已上市产品而另一个单苗尚未上市时，则根据其创新程度（具体见下文），确定二联苗申报和开展临床试验应具备的条件。

**（1）属于境内已上市疫苗的：**可直接申报二联苗的临床试验；单苗若拟单独申请上市，其临床试验可同时申报。二联苗和单苗的临床研究可同期开展。

**（2）属于境内未上市境外已上市的：**同时申报单苗和二联苗的临床试验，需先行开展单苗的试验；可于单苗申报注册并经技术评价（可未完成临床试验现场核查、生产现场核查、注册检验）后开展二联苗临床试验。

**（3）属于改良型或创新型疫苗的：**需先行申报和完成单苗临床试验。其中改良型疫苗可于申报注册并经技术评价（可未完成临床试验现场核查、生产现场核查、注册检验）后申报二联苗临床试验；创新型疫苗需先完成单苗的注册上市后申报二联苗临床试验。

### 2. 两个及以上单苗未注册上市的

如果多联苗含有2个研发者未上市的单苗成分时，则以创新程度最高的单苗确定申报临床试验的具体条件。超过2个以上单苗为研发者未注册上市产品的，申报临床试验的要求进一步提高。总之，只要有一个单苗不能获得或预期不能获得安全有效性数据的，则不支持多联疫苗的临床申报。

综上，为鼓励多联苗的研发，可在单苗尚未完成注册的情况下申报多联苗甚至同期开展临床试验，但研发者需充分考虑研发的整体风险，尤其是单苗最终未能达到安全有效性标准时，相应的多联苗临床试验会被暂停。研发者应自行评估相关风险和制定适宜的临床研发和申报计划。同理，建议从联合程度相对较低的疫苗开始研发，逐步研发联合程度更高的疫苗。

# 四、临床试验设计

联合疫苗的临床试验与其他疫苗一样首先应遵循《药物临床试验质量管理规范》和疫苗临床研究相关指导原则的通用要求。由于多联疫苗含多个抗原，临床研究中对于安全性和有效性的也存在更多需要考虑之处。

## （一）通用考虑

由于疫苗通常用于健康人群，多数为健康儿童或婴幼儿，一般情况下其各阶段临床试验选择健康受试者，因此在疫苗的临床试验中，对安全性方面的考虑和风险控制的要求应高于治疗性药物，实行最严格的管理制度，坚持风险管理和全程管控。

### 1. 总体设计

多联疫苗的临床试验应为随机、对照、盲法的设计。

**对照：**多联疫苗可选择以单苗为对照疫苗，也可选择已上市的相同多联苗为对照疫苗。对照疫苗一般应选择原研产品；选择非原研产品时应提供充分、合理的依据。以单苗为对照的，如具有各单苗联合接种的研究数据支持，对照组可选择联合接种（同时在不同部位接种各单苗），否则应考虑分别设立单苗对照组。

**盲法：**因单苗接种对照组会在不同部位接种单苗，不利于保持盲态，因此多联苗组应在相应部位接种空白对照或安慰剂，保证所有受试者接种部位、剂次和剂量完全相同。建议使用空白对照而非安慰剂对照或阴性对照，更利于客观的进行安全性的绝对评价。

**免疫程序：**当单苗具有一定创新性，免疫程序统一程度不高时，应充分开展免疫程序的探索（以及抗原量、佐剂的等探索），以确保单苗自身获益风险比最大化。直接使用同类疫苗的免疫程序既不利于单苗的充分研发，也不利于后续多联疫苗的合理评价。各单苗如基础免疫和加强免疫程序均相同，多联苗临床试验应包括加强免疫的研究，即涵盖可接种的最后一剂次。

**人群选择：**一般应与单苗的目标人群一致，即覆盖所有可能接种单苗的人群。常规应以易感人群作为主要评价人群。

### 2. 安全性研究

**安全性观察模式：**多联疫苗临床试验的安全性观察及分析的相关要求同常规疫苗，监测模式上采取接种后30分钟现场留观、灭活疫苗0~7天/减毒活疫苗0~14天开展主动监测、8/15~30天主动和被动相结合的模式监测、全程免后随访至6或12个月等。

**安全性信息记录**：不良事件发生例数、例次的基本定义及计算方式同常规疫苗，但由于多联疫苗临床试验受试者存在不止一个接种部位，可能于各部位同时发生相同症状的局部不良事件，例次的计数原则与单苗存在区别。建议客观的按照不同部位分别计入例次，更有利于发现多联苗可能具有的安全性优势（如总例次数的减少）。

**安全性分析框架：**分析内容包括总体不良事件、总体不良反应、全身/局部不良反应、与疫苗无关的不良事件、单个症状（体征、疾病、临床指标）、严重不良事件等，有的还需关注特定的不良事件；减毒活疫苗还可能发生抗原成分间的重组反应，致毒力回复，故在单苗和多联的临床试验中均应开展排毒研究，关注毒力回复和变异的趋势。安全性分析指标一般包括发生频率和严重程度。安全性分析维度则一般包括总体（全程）、发生时间、发生剂次、亚组人群等。

**统计学分析：**同常规疫苗，安全性一般缺乏前置的研究假设和样本量估算的支持，故安全性分析中统计学检验并不能直接用于推断和产生结论，而是作为评价的辅助手段。因此即便多联疫苗和单苗联合接种的安全性存在统计学差异，也应结合这种差异的临床意义和耐受性综合考虑，对联合后安全性的影响进行合理的评价。

### 3. 有效性研究

现有研究提示，联合后某个单苗抗原成分可能会对其它一种或多种抗原成分起到抑制或增强的作用。

#### （1）免疫原性

免疫原性比较研究的目的在于证明多联疫苗接种后各成分疫苗的免疫应答与联合接种单苗后的免疫应答之间是否有显著区别。当单苗已具有免疫原性替代终点时，多联疫苗的相应抗原可基于免疫原性进行有效性的评价；若单苗尚无免疫原性替代终点，多联疫苗也可基于适宜的免疫原性指标进行桥接研究进而评价有效性。因此鼓励在单苗的研发中确认免疫原性替代指标或明确与保护具有相关性的免疫原性评价指标。

临床试验方案应明确免疫应答差异的可接受程度，同时考虑检测方法和受试者的内在变化的影响，合理确定免疫原性非劣效标准。

使用免疫原性替代终点进行有效性评价时，应为经过充分验证的和临床证明的可以保护该疾病的免疫原性指标及其保护性阈值。缺乏免疫原性替代终点时，可根据单苗的研发基础合理选择免疫原性评价指标进行桥接。以抗体为例，一般来讲血清阳转对于临床保护性只起一定的参考作用，应同时开展抗体水平（GMT或GMC）的比较；同时也需注重抗体的功能性而不仅仅是抗体的量。

#### （2）临床终点

一般来说，若单苗需以临床终点确认其有效性时，应先行在单苗研发阶段开展保护效力试验，并探索合理的免疫原性替代终点或免疫原性桥接指标。但当单苗保护性试验既未获得免疫原性替代终点也未明确免疫原性桥接指标时，或是研发者拟采取最保守的策略继续以保护性研究验证多联苗有效性时，多联苗的临床研究会涉及临床终点及评价。

疫苗保护效力试验的相关要求和指导性意见可参考相关指导原则的通用要求。需认识到多联疫苗开展保护效力试验进一步提高了设计的复杂性和实施的难度。需强调的是，随着单苗的临床或药学变更，加之监管标准提高的内在需求，既往采用免疫原性替代终点评价上市的单苗未来可能需采用临床终点进行评价，多联苗的评价要求可能相应改变。

## （二）特殊考虑

### 1. 仅一个单苗未注册的

基于相同免疫程序研发多联苗是最为常见的情形，仅一个单苗未注册上市（以下简称“在研单苗”）且为境内已上市疫苗的，可分为在研单苗拟上市和不拟上市两种可能的情形，其研究设计应分别考虑。

#### （1）单苗拟上市

如采取同期研发的方式，则是将在研单苗与已上市单苗的比较、单苗联合接种与多联苗接种的比较两个研究合并后同时开展。其中在研单苗与上市单苗的比较，为了最大限度减少联合接种因素的干扰，建议采取单独接种的方式，以客观评价两者安全有效性的可比性。因此同期研发时的关键性注册试验最好设置：已上市单苗单独接种组、在研单苗单独接种组、在研或上市单苗联合接种组、多联疫苗接种组等4个试验分组，研究的总体复杂性较高。

在早期试验中，为了减少受试者直接接种多联苗（含在研单苗成分）的风险，应采取在研单苗单独接种组先行入组的设计，待完成早期试验初步积累其安全性数据后再行入组多联苗接种组；多联苗和在研单苗的关键性注册研究可同步开展。

#### （2）单苗不拟上市

在提供在研单苗成分具有或者能够合理预期具有安全性的数据支持条件下，可仅设置联合接种组（均采用上市单苗）、多联疫苗接种组两个试验分组，通过比较探索多联苗与上市单苗联合接种的安全有效性是否具有可比性。

为确保研究的整体风险可控，鼓励至少制备出满足早期临床试验的在研单苗以直接获得初步的安全有效性数据。

### 2. 两个及以上单苗未注册上市的

若研发者有两个及以上单苗未上市的，以均属境内已上市疫苗为例，若拟申报注册上市，则需采取整体研究更多试验分组、早期研究中在研单苗先行接种的研究设计。若在研单苗不拟上市，则需提供其具有或者能够合理预期具有安全性的数据支持。

### 3. 仅用于加强免疫

分为研发者已有相应的基础免疫疫苗和尚无基础免疫疫苗两种情况。

#### （1）已有基础免疫疫苗

其相应各单苗成分的安全有效性具备合理预期的基础，加强免疫的受试者可直接接种多联苗。试验分组可采用多联苗加强免疫组与上市单苗联合接种加强免疫组的比较设计。但需首先考虑两组间加强免疫前的可比性，包括人口学特征、加强免前免疫状态等。鉴于加强免前的免疫状态可能受到基础免前免疫状态、基础免疫所用疫苗的影响，故建议采取自基础免疫开始入组受试者的研究设计，两组受试者使用相同的疫苗完成基础免疫以确保上述因素的组间均衡性；同时加强免疫时联合接种对照组使用与基础免疫相同的疫苗。若采用自加强免疫开始入组的设计，则需考虑上述人口学特征、加强免前免疫状态（以及基础免前免疫状态、基础免疫所用疫苗）等加强免前不受控因素对组间均衡性的可能影响，并相应考虑增大样本量予以弥补。

#### （2）尚无基础免疫疫苗

同前，需首先提供在研单苗或成分具有或者能够合理预期具有安全有效性的数据支持。

## （三）评价标准的调整

鉴于多联疫苗临床研究的复杂程度和实施难度，在单苗研发充分的前提下，多联苗可减少部分评价要求，但安全性研究、有效性中的持久性研究要求不应减少。

**评价项目：**例如，Sabin株脊灰灭活疫苗（sIPV）苗若完整的开展了与野毒株的交叉中和试验，那么含sIPV的多联疫苗则无需再开展该试验；同理，与脊灰减毒活疫苗（OPV）的序贯接种研究也可不再要求；又例如肺炎球菌结合疫苗的试验同时进行了IgG与OPA抗体的检测，若研发相关多联疫苗，则可仅选择其中一种抗体进行免疫原性比较。

**非劣效标准：**一般来说，多联苗与单苗免疫原性比较的非劣效标准应与单苗单独研发的研究相同，但联合后单苗成分可能会对其它抗原成分起发挥抑制作用可能是不可避免的。如果多联疫苗某成分不能达到单苗与单苗比较的非劣效标准但仍能达到保护性界值时，应提供相应的持久性数据以辅助评价这种差异能否接受，并结合临床获益综合评价。但这种情况下，后续研发的相同多联疫苗不宜再选择上述多联苗作为对照。

**免疫原性与保护性的相关性：**鉴于多联疫苗的抗原成分多、待评价的临床终点复杂、临床设计更为复杂，研究内容过多可增加设计和实施难度和导致失败，故各抗原成分的免疫原性与保护性相关性的研究应主要依赖于单苗的研发中开展探索，多联疫苗临床试验可不涉及。

# 五、上市后的变更

多联疫苗完成研发注册上市后也存在改变的需求。随着疫苗上市后更大规模的应用于真实世界的数据积累和基础研究的进展（如免疫原性保护性阈值变化等），部分单苗自身可能需要进行调整，如生产工艺、抗原及佐剂含量及配比、疫苗类型等。单苗变更的相关要求参见药学和临床的相关变更指导原则，多联苗则视相应单苗的变更分类进行要求：单苗变更不需临床验证的，多联苗可相应直接变更，不需开展临床验证；单苗变更需临床验证的，多联苗需申报开展临床试验。多联苗需临床验证时，对单苗的临床研究基础要求同前述首次研发的要求，即视变更后单苗的创新程度确定申报多联苗临床试验的进度要求。

# 参考文献

1. 原国家食品药品监督管理局. 联合疫苗临床前和临床研究技术指导原则. 2005
2. U.S. FDA. Guidance for Industry for the Evaluation of Combination Vaccines for Preventable Diseases: Production, Testing and Clinical Studies. 1997
3. Stanley AP,Walter AO,Paul AO,et al. Vaccines. Sixth Edition. 2016
4. 原国家食品药品监督管理局. 疫苗临床试验技术指导原则. 2004
5. 国家药品监督管理局. 预防用疫苗临床可比性研究技术指导原则. 2019
6. 国家药品监督管理局. 预防用疫苗临床试验不良事件分级标准指导原则. 2019
7. 国家药品监督管理局. 预防用含铝佐剂疫苗技术指导原则. 2019
